# Endoscopic Urinary Diversion As Initial Management of Symptomatic Obstructive Ectopic Ureter in Infants

**DOI:** 10.3389/fped.2017.00208

**Published:** 2017-09-29

**Authors:** Ruben Ortiz, Alberto Parente, Laura Burgos, Jose Maria Angulo

**Affiliations:** ^1^Hospital General Universitario Gregorio Marañón, Madrid, Spain

**Keywords:** ectopic ureter, obstruction, infants, meatus ureter, endourology

## Abstract

**Aim:**

Definitive surgery of ectopic ureter in infants is challenging. We propose an endoscopic urinary diversion (EUD) as a novel surgical technique in the initial management of symptomatic obstructive ectopic ureter.

**Patients and methods:**

Sixteen obstructive ectopic ureters (14 patients) were initially treated by EUD between 2006 and 2015. All patients had urinary tract dilatation worsening at preoperative US scans and at least two febrile urinary tract infection (UTI) or urinary sepsis despite antibiotic prophylaxis. Ectopic ureter was confirmed by cystoscopy. When ectopic meatus was not found, EUD consisted in the creation of a transurethral neo-orifice (TUNO) performed by needle puncturing of the ureterovesical wall, under fluoroscopic and ultrasound control. If ectopic meatus was identified in the posterior urethra, “intravesicalization procedure” was done opening the urethral–ureteral wall to create a new ureteral outlet into the bladder.

**Results:**

EUD was done at a median age of 3.5 months (0.5–7) with median follow-up of 48 months (24–136). TUNO was performed in six patients and “intravesicalization” in eight patients. Significant differences were observed in ureteral diameter and anteroposterior pelvis diameter before and after endoscopic treatment (*p* < 0.005). Initial renal function was preserved in all cases. Postoperative complications were UTI in four patients and TUNO stenosis in one patient, treated by endoscopic balloon dilation. Definitive treatment was further individualized in each patient after 1 year of life.

**Conclusion:**

EUD is a feasible and safe less-invasive technique in the initial management of symptomatic obstructive ectopic ureter. It allows an adequate ureteral drainage preserving renal function until definitive repair if necessary and does not invalidate other surgical options in case of failure or future definitive treatments.

## Introduction

The ureteral ectopia occurs in approximately 1/2,000 newborn. The ectopic ureter is characterized by ending outside its normal opening in the trigone and always at one point along the embryonic mesonephric system path. 80% of patients is associated with a nephroureteral duplicated system, where the upper pole is often dysplastic or with minimal function (1–4). However, this ectopic ureteral ending is usually obstructive with the consequent risk of severe urinary tract infection (UTI) or loss of renal function if present.

Definitive surgery for symptomatic obstructive ectopic ureter in small infants is challenging (5–7). Traditional approach with ureteral reimplantation has shown complications due to the small size of the bladder and the large size of the dilated megaureter (8, 9). For this reason, temporary urinary diversions could be indicated during first months of life (7, 10), but are not exempt of complications. External ureterostomies may present problems such as infections, skin irritations, and stenosis. In addition, parental tolerance is usually low demanding early closure (10–12). Percutaneous nephrostomies could be done with external tubes but have limited durability in small infants.

Other procedures have been proposed to avoid bladder surgery in cases of huge obstructive ectopic ureters. Ipsilateral ureteroureterostomies (13) have been done with acceptable results even through minimally invasive techniques (14–16). In the same way, upper urinary tract approach (lumbar ureteroureterostomies or upper pole heminephrectomy if indicated) could be done laparoscopically, but all these procedures are technically demanding in small infants (5, 17, 18). Other authors advocate performing a refluxing megaureter reimplantation through a small laparotomy, as a temporary internal urinary diversion, with definitive ureteral reimplantation months later (19–21).

We propose to create an endoscopic urinary diversion (EUD) in the initial management of symptomatic ectopic obstructive ureter in infants. The aim of this novel surgical technique is to create a temporary internal urinary diversion to release obstruction, avoiding renal function impairment if present and severe UTIs until definitive surgery is done. We report our experience with this minimally invasive approach and analyze the results and outcomes in a group of patients.

## Patients and Methods

A retrospective review of patients with ectopic obstructive ureter treated between 2006 and 2015 was conducted. Sixteen consecutive obstructive ectopic ureters (14 patients) were initially treated by EUD in this period. In other three patients, this approach was not feasible, in two girls with ectopic ureter ending in vagina and one boy in seminal vesicle. In those patients, ectopic ureter ran away from the bladder without protruding in it during cystoscopy, being the transvesical puncture unsuccessful. Then, video-assisted ureteroureterostomy was performed in those cases.

Twelve patients had prenatal diagnosis of hydronephrosis and received antibiotic prophylaxis since birth; the other two patients were diagnosed after febrile UTI. Twelve patients had unilateral ectopic ureter (eight left side/four right side) corresponding to the upper pole unit in duplicated reno-ureteral systems. The remaining two cases were bilateral ectopic ureters in non-duplicated kidneys.

The presumptive diagnosis of ectopic obstructive ureter was established in all cases attending to sonographic images, renal scintigraphy scans, and voiding cystography. In eight patients, URO-MR was necessary to achieve diagnosis.

US was used to measure the diameter of pelvis, calyces, distal ureter, and the characteristics of renal parenchyma. Hydroureteronephrosis grade was defined according to the guidelines of the Society of Fetal Urology. It was done at birth (in cases of prenatal diagnosis), at 1 month of life, and then every 3 months. Preoperative ultrasonography showed urinary tract dilatation worsening with grade IV hydronephrosis in all cases and bilateral grade III hydronephrosis in a girl without duplicity at time of treatment. None had VUR to the affected ureter.

Mercaptoacetyltriglycine (MAG-3) renal scans revealed obstructive pattern in all ectopic ureters, with severe loss of function of the upper pole moiety (UPM) in four patients (less than 15% of the split renal function), and mild-to-moderate hypofunction in four (less than 40% of the split renal function). The remaining renal unities (*n* = 8) preserved normal function.

Ectopic ureter was always confirmed by cystoscopy. It was indicated in those patients with high suspicion at the imaging tests, with urinary tract dilatation worsening at the US scans and breaking through UTIs despite antibiotic prophylaxis. Renal function or split function of the affected upper pole did not influence in the surgical indication since only symptomatic patients with at least two febrile UTI or urinary sepsis were treated. Furthermore, two patients with severe loss of function of the UPM were treated during urinary sepsis with uretero-pyonephrosis.

When ectopic meatus was not found or was localized in the vagina or in urethrovaginal wall, EUD of the ectopic megaureter consisted in the creation of a transurethral neo-orifice (TUNO) performed by puncturing of the vesicoureteral wall, under fluoroscopic and ultrasound control.

If the obstructive ectopic meatus was identified in the posterior urethra, “intravesicalization procedure” was done, by opening the urethral–ureteral wall with electrocautery to create a ureteral outlet into the bladder.

Clinical data, ultrasonography images, scintigraphy scans, and outcome were preoperatively and postoperatively analyzed. Statistical analysis was performed with IBM SPSS statistics 20.0, using Chi-square test for qualitative variables, and Student’s *t*-test and Wilcoxon test for quantitative analyses.

### Surgical Technique

#### Creation of a TUNO

Under general anesthesia and with antibiotic prophylaxis, a cystoscopy (with a 9.5-Fr Storz cystoscope with 5-Fr working channel) is done.

When ectopic meatus is not found, bladder is punctured where the ectopic ureter protrudes with a 3-Fr needle under direct cystoscopic vision and ultrasound guidance, performing an orifice distal to the trigone. In females, we try to locate the ectopic meatus in the vagina or in the urethrovaginal wall, and in case of finding, high-pressure retrograde pyelography is done to facilitate the posterior vesicoureteral puncturing.

**Figure 1:**
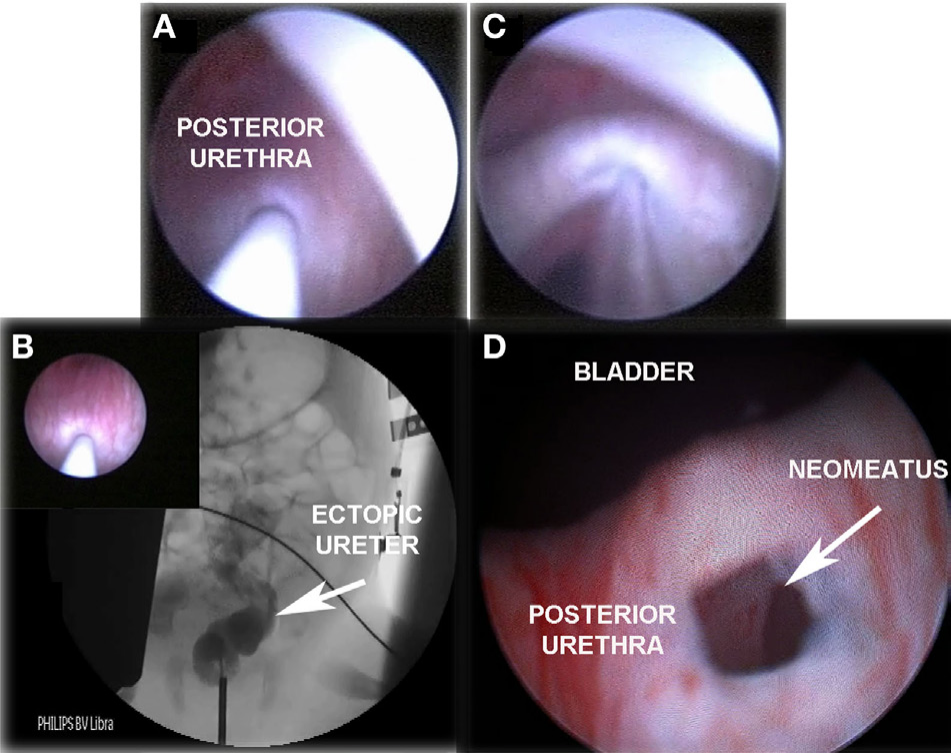
“TUNO” under radioscopic control and postoperative control 3 months later. **(A)** Puncturing of ectopic ureter. **(B)** Retrograde uretero-pyelography. **(C)** Balloon dilatation of the puncturing point creating the neomeatus. **(D)** Neomeatus 3 months later. TUNO, trans-urethral neo-orifice.

**Figure 2:**
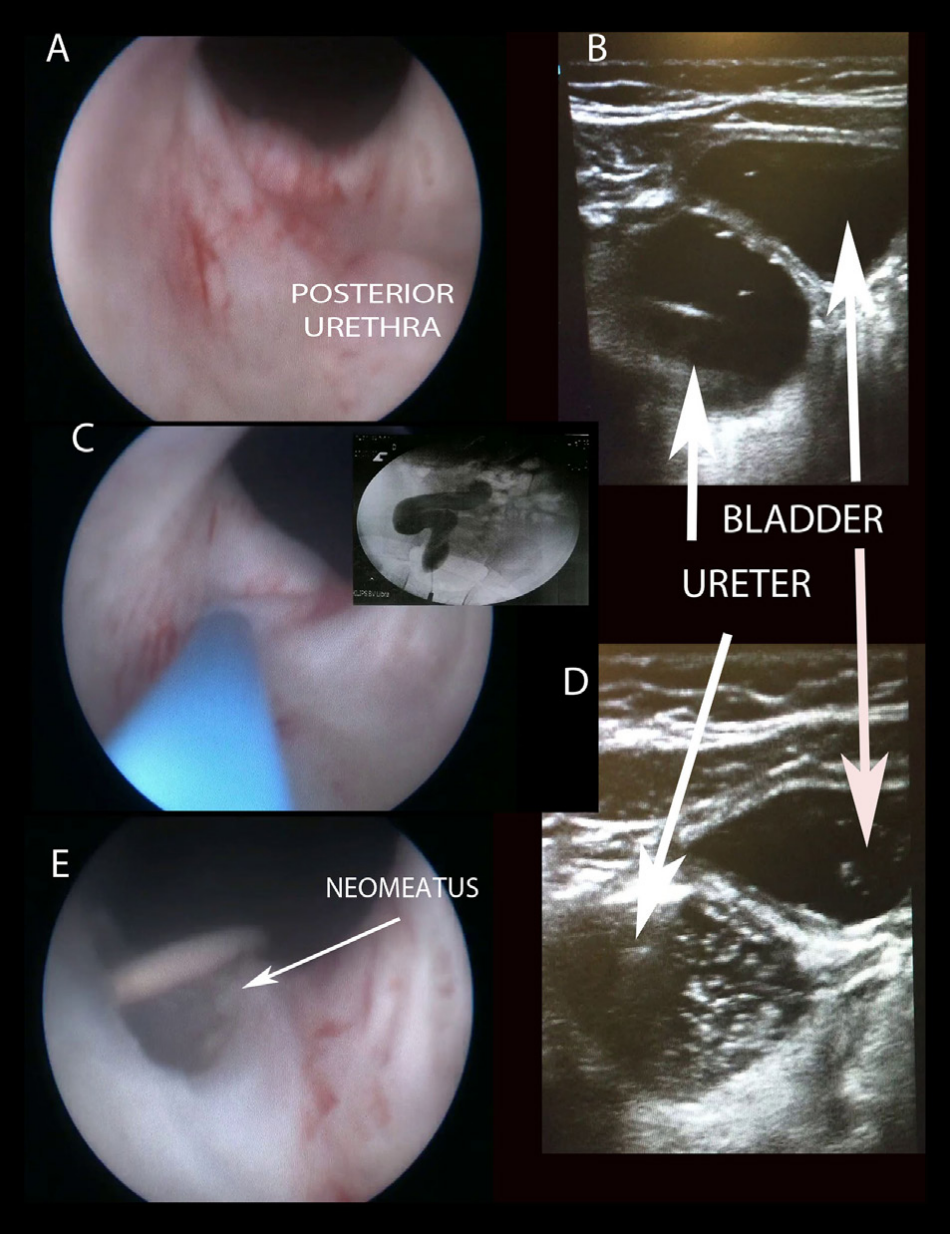
Creating “TUNO” under US and radioscopic control. **(A)** Cystoscopic view of posterior urethra, where ectopic ureter bulges. **(B)** Puncturing vesicoureteral wall under US control (note echogenic guide wire inside the ureter). **(C,D)** Retrograde uretero-pyelography. **(E)** Final appearance of neomeatus with double J stent. TUNO, trans-urethral neo-orifice.

If urine output is observed, radiological contrast is introduced through the needle performing retrograde pyelography under fluoroscopic vision. Then, a hydrophilic flexible guidewire (0.014″ or 0.018″ Terumo^®^) is passed through the ectopic ureter followed by a 5-mm high-pressure balloon (RX Muso, Terumo™), which is inflated to its nominal pressure (14 atm) creating the neomeatus. Neomeatus edges are coagulated with monopolar electrocautery to prevent early closure, and finally a double J ureteral stent (3-Fr, 8–12 cm long, Sof-Flex Multi-Length Ureteral Stent Cook Urological Incorporated™) is left in place for 3–4 weeks (Figures 1 and 2).

#### “Intravesicalization” Procedure

**Figure 3:**
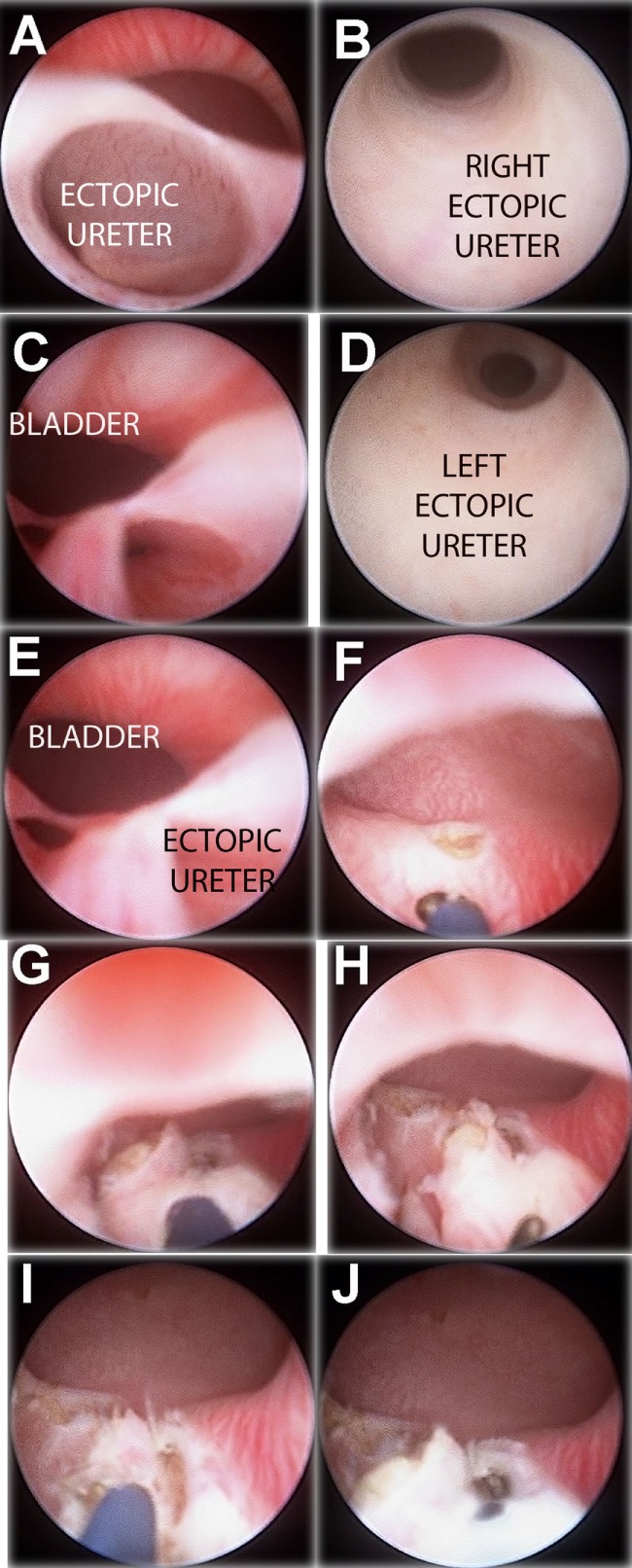
“Intravesicalization procedure” of bilateral ectopic ureters in a girl with anorectal malformation. **(A)** Right ectopic ureteral meatus. **(B)** Ureteroscopy of right ureter. **(C)** Left ectopic ureteral meatus. **(D)** Ureteroscopy of left ureter. **(E)** Both ectopic meatus localized at urethra. **(F,G)** “Intravesicalization” of right meatus. **(H,I)** “Intravesicalization” of left meatus. **(J)** Final result.

When ectopic meatus is located at the posterior urethra, a 4-Fr ureteral catheter is inserted to perform a retrograde pyelography. After pyelography, urethral–ureteral wall is opened with monopolar electrocautery to create a ureteral opening into the bladder. The new ureteral outlet should be on the bladder neck or above to ensure continence, but away from the trigone to avoid injuring the lower pole ureter. We try to perform a limited opening, preserving a bladder mucosal flap above that could partially cover it during bladder filling phase (Figure 3).

### Follow-up

Patients maintained antibiotic prophylaxis and were controlled clinically and with US scan 1 month after EUD and then every 3 months. MAG-3 renal scans and VCUG were performed at the sixth postoperative month.

At 1 year of age, treatment strategy was individualized according to the parents. If UTI occurred during follow-up, definitive surgery was proposed:

– In patients with secondary VUR and renal function preserved, single endoscopic treatment of reflux with subureteral Macroplastique^®^ (*polydimethylsiloxane*) injection or ureteral reimplantation was offered.
– In cases of non-functioning upper pole, heminephrectomy was indicated.

On the other hand, patients who remained asymptomatic were long-term evaluated clinically and with US scans every 6 months. In those cases of asymptomatic secondary VUR, single attempt of endoscopic anti-reflux treatment was offered, with expectant follow-up not performing VCUG routinely (Table 1).

**Table 1:** Flow chart after endoscopic urinary diversion (EUD) in obstructive ectopic megaureter.

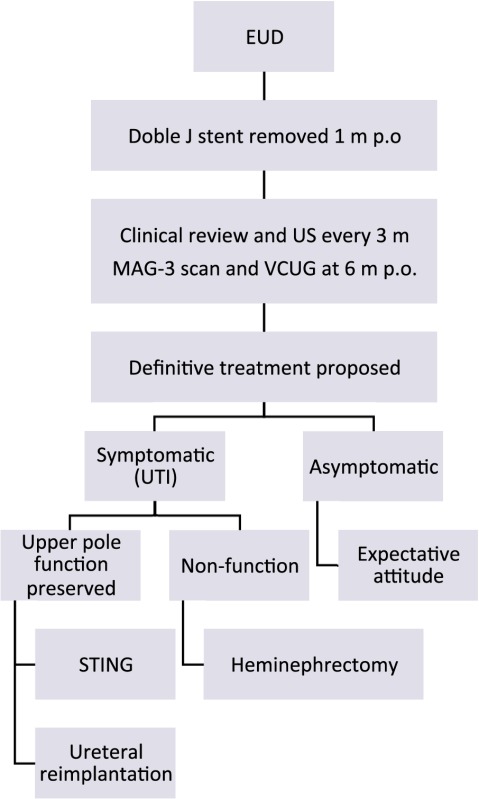

## Results

A total of 16 ectopic ureters in 14 patients were treated from 2006 to 2015, with a median follow-up of 48 months (24–136).

Endoscopic urinary diversion was performed at a median age of 3.5 months (0.5–7), with a median operative time of 35 min (15–70). TUNO was performed in six patients and “intravesicalization” in eight patients. The length of hospital stay was 24 h in all patients, except in two in whom the endoscopic approach was done during hospital admission for urinary sepsis and uretero-pyonephrosis, requiring medical assistance after the procedure during 3 and 9 days, respectively. In one infant, the “intravesicalization” procedure was performed on an outpatient basis. No intraoperative complications were observed.

Attending to the US findings, important improvement was observed in uretero-hydronephrosis, with statistically significant differences in ureteral diameter and anterioposterior pelvis diameter before and after endoscopic treatment (*p* < 0.005) (Table 2). Postoperative MAG-3 renal scan revealed resolution of the obstruction in all cases, preserving initial renal or split renal function.

**Table 2:** Renal US findings in patients with endoscopic urinary diversion.

	Preoperatory	Late p.o. US	*p* Value (Wilcoxon test)
Median distal ureter diameter, mm (range)	17 (30–10)	9 (14–5)	<0.005
Median anterioposterior pelvis, mm (range)	16.5 (27–11)	7 (17–4)	<0.005

Postoperative complications were UTI in four patients and early TUNO stenosis in one who required endoscopic balloon dilatation. It was suspected after an early UTI presented 3 weeks later, with uretero-hydronephrosis worsening. Cystoscopy showed severe neo-orifice stenosis, requiring balloon dilatation with a 6-mm high-pressure balloon, being the patient free of symptoms since then.

Secondary VUR was confirmed in 11/16 cases, but was asymptomatic in most of them. A single STING procedure was offered in all patients with secondary VUR, being done in nine cases, eight of them with non-symptomatic reflux. Subsequent follow-up was done with periodical clinical evaluation and US scans every 6 months, performing VCUG only in cases of persistent urinary tract dilation or symptoms.

Finally, four patients underwent definitive treatment that consisted in three heminephrectomies and one ureteral reimplantation. Heminephrectomies were done in those cases of non-functioning UPM and symptomatic UTI after EUD (two in TUNO group and one in “intravesicalization” group). Ureteral reimplantation was performed in one case of persistent secondary VUR; this reflux was low grade and symptom-free but parents preferred definitive surgery repair.

The remaining patients (10/14) maintained asymptomatic during follow-up with progressive improvement of the urinary tract dilatation in the US, so no further treatments were needed. Continence was evaluated in all of them during long-term, and all patients over 3 years old referred to be dry (actually only one patient is 2-year olds and is now acquiring continence).

Results, complications, and treatments are summarized in Table 3.

**Table 3:** Clinical data, complications, and treatments in patients with endoscopic urinary diversion.

Age	Hydronephrosis grade pre/post	APD pre/post (mm)	RUD pre/post (mm)	Renal scintigraphy (UPM)	Postoperative urinary tract infection (UTI)	2° VUR	Reoperation	Definitive treatment
**Transurethral neo-orifice (TUNO) group**								

3 months	IV/II	23/8	27/4	Severe loss of function	1	No	No	Heminephrectomy
2 months	IV/III	20/10	21/9	Severe loss of function	0	Yes	STING	No
16 days	IV/IV	18/14	15/15	Severe loss of function	1	Yes	No	Heminephrectomy
2 months	II/II	8/9	20/17	Hypofunction	1 early	No	“TUNO” re-dilatation	No
5 months	IV/II	17/10	17/9	Normal	2	Yes	STING + Circumcision	No
5 months	IV/I	16/7	20/7	Normal	0	Yes	STING	No

**“Intravesicalization” group**								

6 months	IV/II	24/14	18/8	Normal	0	Yes	STING	Ureteral reimplantation
6 months	IV/II	25/12	16/4	Hypofunction	0	Yes	STING	No
4 months	IV/II	26/14	21/9	Severe loss of function	2	Yes	No	Heminephrectomy
7 months	IV/I	16/7	14/7	Hypofunction	0	Yes	STING	No
2 months^a^	Right III/I	12/7	13/6	Normal	0	No	No	No
	Left III/I	13/8	12/5	Normal				
5 months	IV/I	14/8	13/8	Normal	0	No	No	No
2 months	IV/III	30/13	17/7	Hypofunction	0	Yes	STING	No
3 months^a^	Right IV/II	14/5	11/4	Normal	0	Yes	STING	No
	Left IV/II	15/7	12/4	Normal		Yes	STING	No

*APD, anterioposterior pelvic diameter; RUD, retrovesical ureteral diameter; UPM, upper pole moiety*.

*^a^Patient with bilateral ectopic ureters in non-duplicated kidneys*.

## Discussion

Neonates and infants with distal ureteral obstruction are at risk of severe UTI and renal loss function. There is still controversy about the need for a temporary urinary diversion or the possibility of complete surgical correction in early stages of life, with special consideration in the small size of the bladder and its disproportion with the diameter of the obstructed ureter. It is known that there is a significant risk of postoperative complications in these patients (22), so alternate techniques have been proposed.

The objective of the initial treatment in obstructive ectopic ureter with recurrent UTI in infants would be to achieve good urine drainage of the obstructive ureter, thereby decreasing the risk of UTI and preserving renal function if present. Accordingly, temporary urinary diversion has proven to be helpful in achieving these objectives (10). Our goal should be to minimize the morbidity associated with these procedures while reaching the same objectives.

Percutaneous nephrostomies could achieve an immediate hydronephrosis improvement, even though their durability is limited in early infants due to drains twists or removal. Therefore, its usefulness is limited to short periods of time (13). Furthermore, its placement in infants is not easy, being described up to 8% of complications including bleeding requiring transfusion, sepsis, or accidental puncture of the pleura or peritoneum (23).

Cutaneous ureterostomies are especially useful when the goal is to maintain the effect of the urinary diversion for several weeks or months (10). Therefore, it may be considered as the first-line treatment in those infants with a large ureteral dilatation that could interfere with a feasible and safe ureteral reimplantation (20). However, several complications have been described in over 25% of cases, as peristomal skin irritation, infection, or stomal stenosis (11). In some occasions, they are poorly tolerated by parents who do not take care of them adequately (10–12).

To avoid this morbidity, internal urinary diversions have become popular, as proposed by Kaefer et al. (19–21), who perform a refluxing megaureter reimplantation through a small laparotomy during the first months of life until definitive surgery is feasible.

The creation of a “TUNO” or the intravesicalization of ectopic meatus pretends to be a endoscopic mean of relieving severe obstruction, to avoid recurrent UTIs and renal function impairment if present, until a future definitive surgery repair is done (24, 25). Only symptomatic patients with uncontrolled UTIs or in the situation of urinary sepsis were treated. For these reasons, function of the affected upper moiety did not influence in the surgical indication. In our series, four patients were initially treated with a very low split renal function (<15%) of the upper pole affected. In two of them (one baby was 16 day olds), endoscopic approach was performed during hospital admission for urinary sepsis and uretero-pyonephrosis. Renal function did not improve in any of them and finally heminephrectomy was performed in three patients 6–12 months later. However, the internal drainage allowed the child to mature and definitive heminephrectomies were done in safe and better conditions. One patient maintained asymptomatic after endoscopic diversion, so an expectant attitude was decided.

We propose this novel technique that is really minimally invasive, with a short operative time and hospital stay and with minimal postoperative disturbance. Furthermore, the ureteral rest left in the creation of a neomeatus is minimal compared to other techniques (6, 19), probably diminishing risks of future complications.

It is important to have on mind that “intravesicalization” procedure is technically easier than the creation of a TUNO, which requires a higher learning curve in endourology. The visualization of the ectopic meatus and the ureteral tract facilitates its endoscopic approach. However, in the creation of a TUNO, we must intuit the ureter path and the adequate puncture site trough the ureterovesical wall. In this way, it is easy to imagine that the complication rate should be higher for the neomeatus procedure than for the “intravesicalization.” However, the most common complication is not finding the ectopic ureter, probably because the ureterovesical wall is thicker than imagined. In our series, no bladder injuries and no perforations of other viscera occurred. The puncture needle has a very low profile and limited length to avoid these possible complications. Those endoscopic approaches have proven to be safe procedures, with fewer complications than other techniques like the refluxing megaureter reimplantation (21) or the laparoscopic/video-assisted ureteroureterostomy (14).

The most frequent complication observed was secondary reflux. Although it is not a desirable condition, it is easier to manage than obstruction and carries less risk for continued renal injury. However, it was asymptomatic in most cases and did not result in significant morbidity for the patients.

It should be emphasized that this endoscopic procedure aims to be a temporary and minimally invasive internal urinary diversion, preserving renal function if present until a time at which these patients could undergo a definitive repair. However, several patients stayed asymptomatic and with resolution of the urinary tract dilatation during postoperative follow-up and therefore, an expectant behavior was kept as in current treatment protocols for ureterocele (5).

The present communication has obvious limitations considering it is a descriptive retrospective study with no control group and so our results should be compared with the literature.

## Conclusion

This EUD has shown to be a feasible technique in the initial management of symptomatic obstructive ectopic ureter. It is a really minimal invasive procedure for a challenging pathology that presents in small infants, allowing an adequate ureteral drainage in cases of severe obstruction with high risk of uncontrollable UTI or uretero-pyonephrosis. The objective of this technique is to be a temporary internal drainage to control urinary infections, preserve initial renal function, and facilitate the baby to mature until definitive surgery is proposed months later. It is reproducible, safe, and does not invalidate other surgical options in case of failure or future definitive treatments.

## Ethics Statement

This study was carried out in accordance with the recommendations of “Normas de manejo de pacientes pediátricos, Comité Deontológico Hospital Gregorio Marañón” with written informed consent from all subjects. All subjects gave written informed consent in accordance with the Declaration of Helsinki. The protocol was approved by the “Comité Deontológico Hospital Gregorio Marañón.”

## Author Contributions

This paper presents a new technique for the treatment of symptomatic ectopic ureter in the infant. It avoids external urinary diversion and it is a minimally invasive technique with good results. All authors have contributed to the management of patients. RO and AP have developed the paper.

## Conflict of Interest Statement

The authors declare that the research was conducted in the absence of any commercial or financial relationships that could be construed as a potential conflict of interest.
